# Sleep health and its related influencing factors in primary and middle school students in Fuzhou: A large multi-center cross-sectional study

**DOI:** 10.3389/fpubh.2022.924741

**Published:** 2022-08-04

**Authors:** Xingyan Xu, Fuhao Zheng, Yingying Cai, Jie Lin, Zhaonan Zeng, Shichao Wei, Siying Wu

**Affiliations:** ^1^Department of Epidemiology and Health Statistics, School of Public Health, Fujian Medical University, Fuzhou, China; ^2^Office of Academic Research, Fujian Provincial Hospital, Fuzhou, China; ^3^The Second Attached Hospital of Fujian Medical University, Quanzhou, China; ^4^Center for Experimental Research in Clinical Medicine, Fujian Provincial Hospital, Fuzhou, China; ^5^Sleep Medicine Center, Fujian Provincial Hospital, Fuzhou, China

**Keywords:** sleep health, children and adolescents, factors, sleep habits, Fuzhou

## Abstract

**Background:**

This current study set out to investigate the status of sleep health in 7–20-year-old students in Fuzhou and explore the related influencing factors of sleep health.

**Methods:**

A total of 38,467 children and adolescents in Fuzhou were included in the study through a random stratified cluster sampling. Data were collected from May to June 2019, in 18 primary schools and 18 middle schools from nine districts, Fuzhou. Children's parents and adolescents of sampled classes were invited to fill out a series of questionnaires about the performance of the last 6 months (sociodemographic characteristics, sleep-related lifestyle behaviors, and electronic-products usage). Multiple linear regression was carried out to analyze data.

**Results:**

Of the total 40,888 questionnaires we released, 38,467 were valid and effective with the response rate was 94.08%. The age of the surveyed participants was 11.85 ± 3.1, including 20,013 boys and 18,454 girls. The multiple linear regression analysis identified factors associated with sleep health (*p* < 0.05): Boy (coef = 0.073, 95% CI: 0.030–0.115), age (coef = 1.797, 95% CI: 0.224–0.243), key school (coef = 2.069, 95% CI: 0.105–0.193), urban (coef = 0.096, 95% CI: 0.054–0.139), excessive daytime sleepiness (coef = 0.535, 95% CI: 0.432–0.639), unhealthy sleep habits (coef = 0.363, 95% CI: 0.307–0.419), eating before sleep (coef = 0.578, 95% CI: 0.527–0.630), using electronic products in bedroom (coef = 0.074, 95% CI: 0.028–0.121), screen time per day during school (coef = 0.260, 95% CI: 0.235–0.284), frequency of using electronics 30 min before bedtime (coef = 0.150, 95% CI: 0.134–0.166), strained relationship with parents (coef = 0.361, 95% CI: 0.270–0.452), strained relationship with peers (coef = 0.267, 95% CI: 0.171–0.363), excessive homework or learning (coef = 0.189, 95% CI: 0.141–0.237), time for doing homework (coef = 0.266, 95% CI: 0.245–0.287), and mood swings frequently (coef = 1.174, 95% CI: 1.127–1.221) negatively impact sleep health. Sleep alone (coef = −0.204, 95% CI: −0.262–0.147) were the risk factors for sleep health. Furthermore, frequent mood swings was considered the most influential factor on overall variables.

**Conclusions:**

Sleep health is associated with factors covered sociodemographic characteristics, family sleep habits, and routine activities before bedtime. Multiple measures should be taken to improve sleep quality in a targeted manner.

## Introduction

Sleep health is a notable concern during school age and adolescence, with the majority of them reporting poor sleep patterns, including insufficient and inferior quality sleep. Sleep problems are estimated to affect 30–70% of adolescents in Europe and North America and 14 to 68% of students sleeping less than recommended on school days (1, 2). Just half (50.19%) of the children slept for >8 h per day and only one-third of children (35.37%) reported good sleep quality in China (3). Compared with other age groups, healthy sleep is challenged by unique features for elementary and junior high school students: extrinsic factors such as social activities and academic demands (with early school start times) interact with autonomy regarding their sleep schedules that cause irregular sleep patterns (1). Previous research has shown that poor sleep in children and adolescents is associated with a variety of problematic outcomes including reduced life satisfaction (4), increased feelings of anxiety and depression (5), increased diagnosis of diseases [attention-deficit/ hyperactivity disorder (6), obesity (7), and diabetes (8), and increased risk for suicide (9)], underscoring the need to include healthy sleep into students health promotion efforts.

Given the high prevalence of sleep deficiency in children and adolescents, the negative impact on a range of outcomes, and the particular sleep characteristics such as sleep duration, sleep disturbance, and sleep quality, it is important to assess factors influencing sleep health in this age group for improving their sleep quality. Sleep health is multifaceted, and requires adequate duration, high quality, and without disturbance such as difficulty falling and staying asleep (10). However, only a few studies have been conducted on the sleep health of primary and middle school students, and even most of these studies focused on sleep duration with little attention to sleep quality. Besides, comparable data about sleep deficiency in children and adolescents from China is limited and related research in Fujian Province is scarce based on reference literature. Hence, this study will therefore provide such estimates.

In this study, data on sleep collected in school-based surveys of primary and middle school students in Fuzhou, Fujian Province, China, were enrolled to describe the sleep patterns in children and adolescents and to assess the factors influencing sleep health (involving sleep quality, sleep duration, and sleep disturbance). This is a rare large-scale study on the sleep health of primary and middle school students in China, which fills the gap in relevant studies in Fujian Province and provides a theoretical basis for formulating measures to improve the sleep of children and adolescents.

## Materials and methods

### Study design

A cross-sectional, randomized, stratified, multistage cluster sampling methodology was conducted to select 36 schools in Fuzhou, a city divided into 13 districts classified as three regions including five urban Districts, six rural districts, and two county-level cities.

Based on the statistical formula:


$$N=deff*\frac{{\left(Z_{\alpha /2}\right)}^{2}*P*(1-P)}{\delta^{2}}$$

(where *n, p*, and δ are sample size, positive rate, and acceptable error, respectively), supposing sleep disorder prevalence = 4.43% in children (11), significance at α = 0.05 with Z_α/2_ of 1.96, and acceptable error at 0.1 P the sample size was calculated as 8,288.

**Figure 1:**
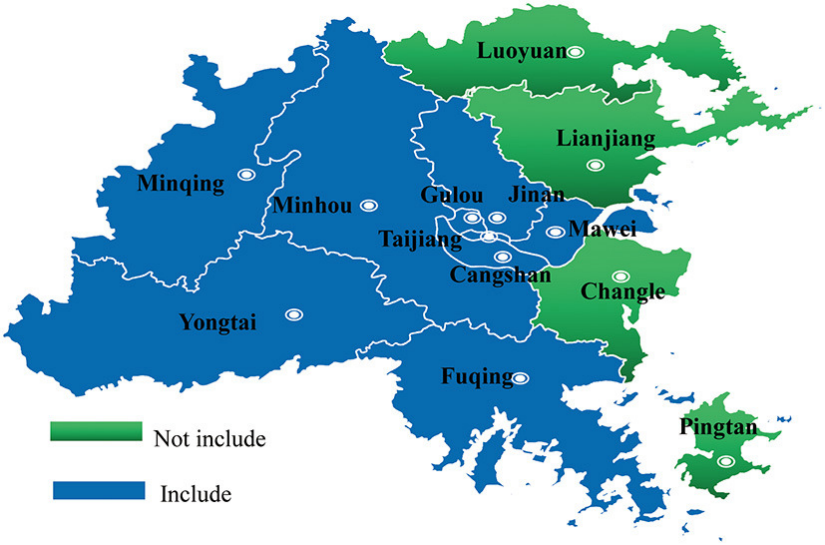
Districts selected in Fuzhou. List of schools: 18 primary schools and 18 middle schools were randomly chosen from 9 selected Districts. Specific list: Yanan Middle School, Yangqiao Middle School, No.2 Primary School, Huaqiao Primary School (Gulou); No.8 Middle School, No.15 Middle School, Shuanghong Primary School, No.5 primary school (Taijiang); No.40 Middle School, No.12 Middle School, Jianshan primary school, No.1 Middle School (Cangshan); Gushan High School, No.20 Middle School, Gucheng Primary School, Jincheng Primary School (Jinan); Langqi Middle School, No.2 High School, Rujiang Primary School, Experimental Primary School (Mawei); Fuqing No.2 Middle School, Yuanqiao Middle School, Shimen Primary School, Jinyin Primary School (Fuqing); Minhou No.6 Middle School, Shangjie Experimental Primary and Middle School, Shangjie Hongfei Primary School (Minhou); Chengguan Middle School, No.3 Middle School, Zhangcheng Primary School, Qingliang Primary School (Yongtai); Minqing Middle School, Chengguan Middle School, Minqing No.2 primary school, Minqing Experimental Primary School (Minqing).

Allowing for non-response (deff = 2), the final intended sample size was set as 16,576. In stage 1, depending on account of regional economic development, five urban districts and four rural Districts were randomly selected from all 13 Districts (five urban Districts, six rural districts and two county-level city) in Fuzhou. In stage 2, two primary schools and two junior middle schools were randomly chosen from the selected Districts (Taijiang District, Mawei District, Canshan District, Fuqing District, Minhou District, Minqing District, Yongtai District, Gulou District, and Jinan District) (Figure 1). In stage 3, classes were selected by the principals in reasoned consideration of the class schedule. Participation was voluntary and anonymous. All students, besides Grade 6, 9, and 12 in chosen schools, were invited to participate in the survey. The students in the presence of a researcher and children's parents completed the self-administered questionnaire about their performance over the last 6 months in normal classroom settings. Data collection occurred from May to June 2019.

### Questionnaire investigation

The sleep quality assessment uses a sleep self-made questionnaire, which is divided into three dimensions: sleep duration, sleep disturbance, and subjective sleep quality (Supplementary Table 1). Sleep health is multi-faceted, including sleep duration, sleep disturbance, and subjective sleep quality.

Sleep duration involves four items (What time do you go to bed on weekdays and weekends? How many hours do you sleep on weekdays and weekends?); Sleep duration scores that vary within a range (4–16). Sleep disturbance includes six items (Do you have nightmares while sleeping? Is there any apnea or awakening while sleeping? Will you wake up more than 2 times a night? Is it difficult to fall asleep during waking up at night? Do you often feel that you can't move when you wake up? Do you feel physical pain during sleep?); The range of sleep disturbance scores varies from 2 to 12. Subjective sleep quality dose consists of three items (Did you have a good sleep? How long have you been sleepless? Do you feel a lack of sleep?); Subjective sleep quality score that varies within a range (1–7). The sum score of these three dimensions is the sleep health score (range: 7–35). The higher the score, the worse the sleep health became.

Reliability tests are tests aiming at improving the precision of a questionnaire. Cronbach's alpha coefficient (α) is used to evaluate the internal consistency. In our survey, Cronbach's α of the present study indicated good reliability of the scale (Cronbach's α = 0.716). Pearson correlation coefficients are used to assess the test-retest reliability. The correlation between overall sleep quality and three dimensions and the distinguishing validity of the scale. Construct validity is measured by Principal components analysis. After standardizing the scores of all items of each scale and establishing a correlation coefficient matrix, Principal components analysis is used to verify construct validity, extracting the common factor through the fourth-order maximum orthogonal rotation. See appendix for the details (Supplementary Tables 2, 3).

In addition, questionnaires dealt with demographic characteristics (sex, age, type of school, area), sleep-related lifestyle behaviors (excessive daytime sleepiness, unhealthy sleep habits, unhealthy eating habits, eating before sleep and sleep alone), electronic-products usage (using electronic products in bedroom, screen time per day during school, frequency of using electronics 30 min before bedtime), and emotional factors (mood swings frequently, a strained relationship with parents, strained relationship with peers) or heavy academic stress (excessive homework or learning, time for doing homework).

The definition of a key school is as follows: key schools, generally under the supervision of the municipal or provincial education authorities, are given priority treatment by various good policies. Key schools will have better educational equipment, quality of students, educational resources, and teaching staff. “unhealthy eating habits” were determined by the following questions: “Do you eat snacks?” (snacking); “Does your eating speed more quickly than other people?” (eating quickly); “Do you continue to eat only a limited variety of foods, rejecting certain types of foods—both familiar and unfamiliar” The answer options were “yes” or “no”. Those who answered yes to a question were defined as having that particular unhealthy eating habit. As this is a study of sleep, there is zeitgeber, so “Eating before sleep” is separated out: “Do you have night meals or snacks within two hours before bedtime?” “excessive homework or learning” is the estimated amount of homework assigned. “Time for doing homework” is the actual time spent on homework. “Strained relationship with parents, a strained relationship with peers” were self-reported by the students and children's parents: “Do you seek support from parents and peers for personal problems” or “Do parents or peers comfort, console, praise, and embrace or express their love through words and actions for you.”

### Statistical analysis

Data collation and analysis were conducted using Epidata 3.0 and SPSS 19.0. Descriptive analysis was performed. Results for continuous variables with normal distributions were described by mean ± standard deviations (SD) and compared using a two-tailed Student's *t*-test or variance analysis. The continuous data were reported as correlation using Pearson's method. Results for discrete variables were displayed as a percentage (%) and distribution differences were examined by the chi square (χ^2^) test. We estimated the strength of the association between overall sleep quality score and predictors by regression coefficient (coef) and 95% confidence interval (CI). Two-sided *p* < 0.05 was considered to be statistically significant.

## Results

### Characteristics of participants

In total 40,888 questionnaires we released, and 38,467 completed questionnaires were collected. Thus, the valid response rate was 94.08%. The demographic and background characteristics of participants are shown in Table 1. The surveyed participants included 20,013 (52.0%) boys and 18,454 (48.0%) girls. Among all subjects in the sample in this study, almost two-thirds of the participants (62.0%) were from key schools. In terms of area, 47.3% lived in urban while 52.7% lived in rural. The mean age was 11.9 ± 3.1years, and the distribution of age was 53.0% for ≤ 12 years, 30.6% for 13–15 years, and 16.3% for ≥ 16 years.

**Table 1:** Characteristics of the study population.

**Variable**	**Characteristics**	***n***	**%**
Sex	Male	20,013	52.0
	Female	18,454	48.0
Type of school	Key school	23,847	62.0
	Others	14,620	38.0
Region	Urban	18,210	47.3
	Rural	20,257	52.7
Age (years)	≤12	20,401	53.0
	13–15	11,782	30.6
	≥16	6,284	16.3

### Comparison of sleep scores by different characteristics

Table 2 showed the results from the comparison of sleep scores by different characteristics. Students aged > 12 years had a higher sleep health score, sleep duration score, sleep disturbance score, and subjective sleep quality score than participants aged ≤ 12 years.

**Table 2:** Comparison of sleep scores by different demographic characteristics.

**Variable**	**Sleep health**	**Sleep duration**	**Sleep disturbance**	**Subjective sleep quality**
Sex		^*^	^**^	
Male	8.36 ± 2.62	3.13 ± 1.03	3.25 ± 1.19	1.98 ± 1.31
Female	8.41 ± 2.52	3.16 ± 0.98	3.29 ± 1.16	1.96 ± 1.18
Age (years)	^**^	^**^	^**^	^**^
≤12	7.31 ± 2.03	2.66 ± 0.78	2.99 ± 1.03	1.67 ± 1.04
>12	9.60 ± 2.59	3.71 ± 0.93	3.58 ± 1.25	2.31 ± 1.37
Type of school		^*^	^*^	
Key school	8.37 ± 2.61	3.14 ± 1.00	3.26 ± 1.18	1.98 ± 1.27
Others	8.41 ± 2.52	3.17 ± 1.00	3.29 ± 1.18	1.96 ± 1.22
Region				^**^
Urban	8.37 ± 2.62	3.15 ± 1.02	3.28 ± 1.22	1.94 ± 1.25
Rural	8.40 ± 2.53	3.15 ± 0.99	3.26 ± 1.14	1.99 ± 1.25

* *p < 0.05;*

** *p < 0.001*.

**Figure 2:**
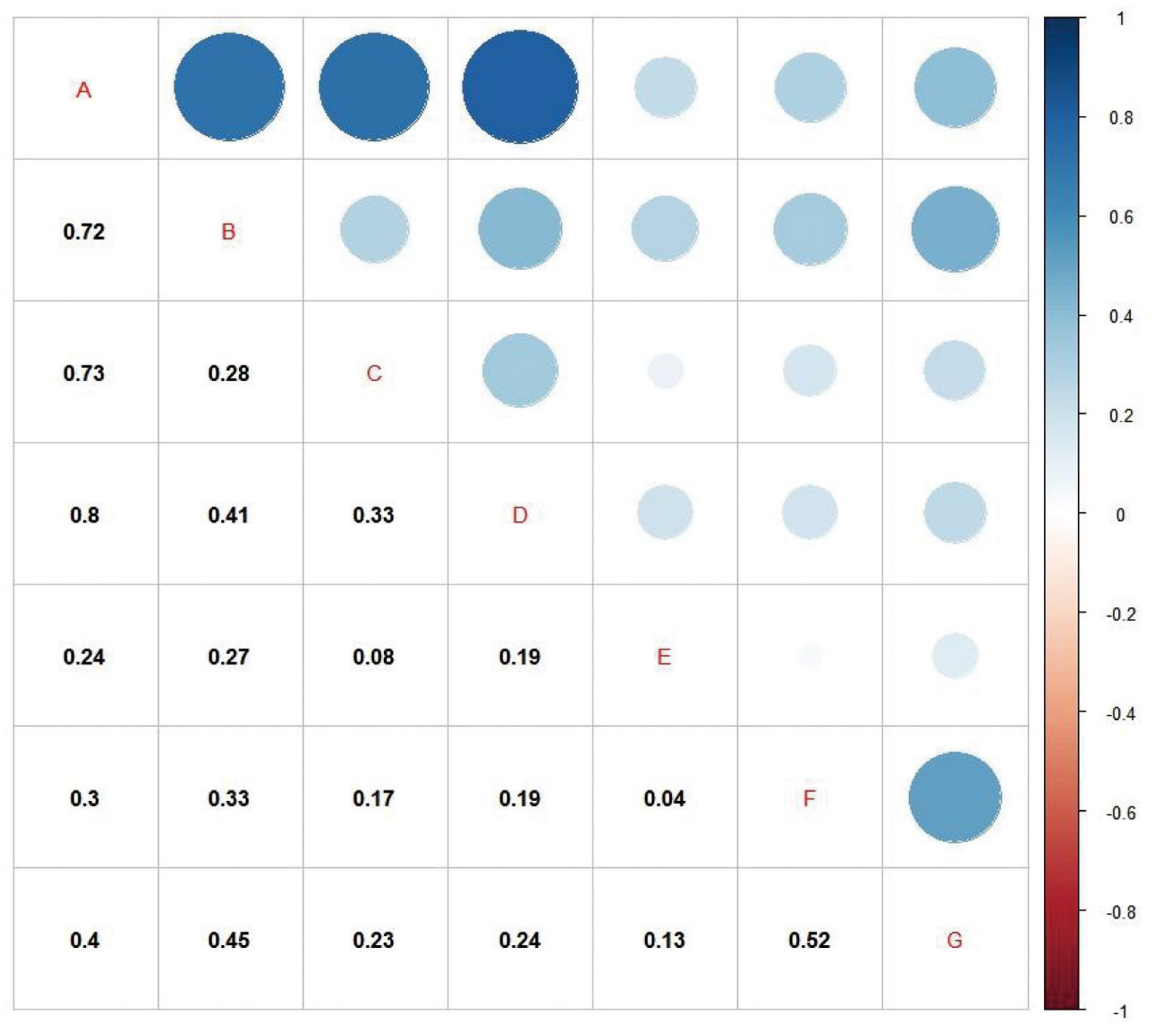
Correlation analysis of factors affecting sleep health. **(A)** Sleep health score; **(B)** Sleep duration score; **(C)** Sleep disturbance score; **(D)** Subjective sleep quality score; **(E)** Time for doing homework; **(F)** Screen time per day during school; **(G)** Frequency of using electronics 30 min before bedtime. All of *p* < 0.001. The color shades and size of the circle indicate the correlation coefficient (*r*) between any two factors.

We investigated the relationship between behavioral and emotional factors and sleep quality by comparing the sleep health score, sleep duration score, sleep disturbance score, and subjective sleep quality score of children according to the presence or absence of behavioral and emotional problems (Table 3 and Figure 2). We found that behavioral factors such as excessive daytime sleepiness, unhealthy sleep habits, unhealthy eating habits, eating before sleep, electronic media use (using electronic products in the bedroom, screen time per day during school, frequency of using electronics 30 min before bedtime) and sleep alone have an unhealthy impact in sleep quality. In terms of emotional factors, participants who suffered from strained relations with parents/peers or heavy academic stress (excessive homework or learning, time for doing homework) had poor sleep quality. And moreover, the sleep health score was significantly higher in the presence of fluctuating emotions.

**Table 3:** Univariate analysis of the factors associated with sleep characteristics.

	***n*** **/%**	**Sleep health**	**Sleep duration**	**Sleep disturbance**	**Subjective sleep quality**
Excessive homework or learning		^**^	^**^		^**^
Yes	26,311/86.4	8.43 ± 2.55	3.17 ± 0.98	3.26 ± 1.16	2.00 ± 1.26
No	12,156/31.6	8.28 ± 2.62	3.10 ± 1.05	3.28 ± 1.20	1.90 ± 1.23
Excessive daytime sleepiness		^**^	^**^	^**^	^**^
Yes	1,692/4.4	9.38 ± 3.15	3.48 ± 1.21	3.62 ± 1.52	2.27 ± 1.45
No	36,775/95.6	8.34 ± 2.54	3.13 ± 0.99	3.25 ± 1.16	1.95 ± 1.24
Unhealthy sleep habits		^**^	^**^	^**^	^**^
Yes	7,340/19.1	9.00 ± 2.79	3.34 ± 1.04	3.43 ± 1.24	2.23 ± 1.39
No	31,127/80.9	8.24 ± 2.50	3.10 ± 0.99	3.23 ± 1.15	1.90 ± 1.21
Unhealthy eating habits		^*^		^**^	^**^
Yes	2,085/5.4	8.52 ± 2.76	3.11 ± 1.03	3.35 ± 1.28	2.06 ± 1.34
No	36,382/94.6	8.38 ± 2.56	3.15 ± 1.00	3.26 ± 1.17	1.96 ± 1.24
Eating before sleep		^**^	^**^	^**^	^**^
Yes	8,382/21.8	8.90 ± 2.89	3.25 ± 1.07	3.47 ± 1.36	2.17 ± 1.38
No	30,085/78.2	8.24 ± 2.46	3.12 ± 0.98	3.21 ± 1.11	1.91 ± 1.21
Using electronic products in bedroom		^**^	^**^	^**^	^**^
Yes	20,110/52.3	8.88 ± 2.65	3.75 ± 1.03	3.38 ± 1.22	2.12 ± 1.31
No	18,356/47.7	7.84 ± 2.37	2.90 ± 0.91	3.14 ± 1.11	1.80 ± 1.16
Sleep alone		^**^	^**^	^**^	^**^
Yes	30,828/80.1	8.53 ± 2.58	3.23 ± 1.00	3.30 ± 1.18	2.00 ± 1.26
No	7,639/19.9	7.79 ± 2.44	2.81 ± 0.93	3.16 ± 1.14	1.82 ± 1.19
Strained relationship with parents		^**^	^*^	^**^	^**^
Yes	2,317/6.0	8.72 ± 2.91	3.10 ± 1.02	3.50 ± 1.35	2.12 ± 1.37
No	36,150/94.0	8.36 ± 2.55	3.15 ± 1.00	3.25 ± 1.16	1.96 ± 1.24
Strained relationship with peers		^**^	^**^	^**^	^**^
Yes	2,075/5.4	9.27 ± 2.96	3.39 ± 1.07	3.59 ± 1.38	2.28 ± 1.41
No	36,392/94.6	8.33 ± 2.54	3.14 ± 0.99	3.24 ± 1.16	1.95 ± 1.24
Mood swings frequently		^**^	^**^	^**^	^**^
Yes	13,002/33.8	9.64 ± 2.84	3.46 ± 1.02	3.70 ± 1.36	2.47 ± 1.42
No	25,465/66.2	7.75 ± 2.16	2.99 ± 0.95	3.05 ± 1.00	1.71 ± 1.06

* *p < 0.05;*

** *p < 0.001*.

Additionally, the correlation coefficient (r) between any two factors was statistically significant (*p* < 0.05). Sleep health score was positively correlated with sleep duration score (*r* = 0.72), sleep disturbance score (*r* = 0.73), and subjective sleep quality score (*r* = 0.8) (Figure 2).

### Multiple linear regression analysis of influencing factors of sleep health

Multiple linear regression was conducted to determine the influence of lifestyle and other factors on sleep health, sleep duration, sleep disturbance, and subjective sleep quality, using “Backward elimination.” We include the factors in the above test and demographic characteristics into the equation of multiple linear regression, which shows items such as demographic characteristics, lifestyle behaviors, and emotional factors that affected sleep health, sleep duration, sleep disturbance, and subjective sleep quality. The collinearity diagnosis found that the VIFs were all < 5, which can be preliminarily considered that the problem of collinearity between independent variables can be ignored.

Boy (coef = 0.073, 95% CI: 0.030–0.115), age (coef = 1.797, 95% CI: 0.224–0.243), key school (coef = 2.069, 95% CI: 0.105–0.193), urban (coef = 0.096, 95% CI: 0.054–0.139), excessive daytime sleepiness (coef = 0.535, 95% CI: 0.432–0.639), unhealthy sleep habits (coef = 0.363, 95% CI: 0.307–0.419), eating before sleep (coef = 0.578, 95% CI: 0.527–0.630), using electronic products in bedroom (coef = 0.074, 95% CI: 0.028–0.121), screen time per day during school (coef = 0.260, 95% CI: 0.235–0.284), frequency of using electronics 30 min before bedtime (coef = 0.150, 95% CI: 0.134–0.166), strained relationship with parents (coef = 0.361, 95% CI: 0.270–0.452), strained relationship with peers (coef = 0.267, 95% CI: 0.171–0.363), excessive homework or learning (coef = 0.189, 95% CI: 0.141–0.237), time for doing homework (coef = 0.266, 95% CI: 0.245–0.287) and mood swings frequently (coef = 1.174, 95% CI: 1.127–1.221) negatively impact sleep health. sleep alone (coef = −0.204, 95% CI: −0.262– −0.147) were the risk factors for sleep health. Furthermore, mood swings frequently was considered the most influential factor on overall variables (Table 4).

**Table 4:** Multiple linear regression analysis of influencing factors of sleep health.

**Variables**	**Sleep health**		**Sleep duration**		**Sleep disturbance**		**Subjective sleep quality**	
	**coef (95% *CI*)**	**VIF**	**coef (95% *CI*)**	**VIF**	**coef (95% *CI*)**	**VIF**	**coef (95% *CI*)**	**VIF**
Boy	0.073	1.021	–	–	–	-	0.078	1.02
	(0.030~0.115)						(0.055~0.101)	1
Age (continuous)	0.234	1.956	0.129	1.95	0.047	1.93	0.058	1.94
	(0.224~0.243)		(0.126~0.133)		(0.042~0.052)	7	(0.052~0.063)	3
Key school	0.149	1.027	0.059	1.027	0.028	1.024	0.063	1.027
	(0.105~0.193)		(0.042~0.075)		(0.005~0.051)		(0.039~0.087)	
Urban	0.096	1.02	0.043	1.02	0.056	1.017	–	–
	(0.054~0.139)		(0.027~0.059)		(0.034~0.079)			
Excessive daytime sleepiness	0.535	1.018	0.166	1.018	0.209	1.015	0.161	1.018
	(0.432~0.639)		(0.127~0.205)		(0.155~0.264)		(0.105~0.217)	
Unhealthy sleep habits	0.363	1.068	0.077	1.085	0.079	1.019	0.205	1.066
	(0.307~0.419)		(0.056~0.098)		(0.051~0.107)		(0.175~0.235)	
Unhealthy eating habits	–	–	0.038	1.03	–	–	–	–
			(0.002~0.073)					
Eating before sleep	0.578	1.019	0.127	1.016	0.231	1.014	0.220	1.018
	(0.527~0.630)		(0.108~0.147)		(0.204~0.258)		(0.192~0.248)	
Using electronic products in bedroom	0.074	1.209	0.056	1.207	0.066	1.392	0.028	1.208
	(0.028~0.121)		(0.038~0.073)		(0.053~0.078)		(0.003~0.053)	
Screen time per day during school	0.260	1.426	0.104	1.424	–	–	0.088	1.419
	(0.235~0.284)		(0.095~0.113)				(0.075~0.101)	
Frequency of using electronics 30 min before bedtime	0.150	1.904	0.064	1.994	0.047	1.905	0.038	1.992
	(0.134~0.166)		(0.058~0.070)		(0.039~0.055)		(0.029~0.047)	
Sleep alone	−0.204	1.168	−0.037	1.161	−0.067	1.157	−0.099	1.168
	(−0.262~−0.147)		(−0.059~−0.016)		(−0.097~−0.037)		(−0.130~−0.068)	
Intense relationship with parents	0.361	1.052	–	–	0.198	1.051	0.136	1.052
	(0.270~0.452)				(0.150~0.245)		(0.087~0.186)	
Intense relationship with peers	0.267	1.058	0.068	1.019	0.114	1.058	0.091	1.058
	(0.171~0.363)		(0.033~0.103)		(0.064~0.164)		(0.039~0.143)	
Excessive homework or learning	0.189	1.116	0.046	1.115	–	–	0.120	1.113
	(0.141~0.237)		(0.028~0.064)				(0.095~0.146)	
Time for doing homework	0.266	1.131	0.124	1.131	0.014	1.101	0.130	1.131
	(0.245~0.287)		(0.116~0.132)		(0.003~0.025)		(0.119~0.142)	
Mood swings frequently	1.174	1.102	0.131	1.097	0.487	1.099	0.557	1.101
	(1.127~1.221)		(0.114~0.149)		(0.462~0.511)		(0.532~0.583)	

## Discussion

Healthy sleep is essential for children's and adolescents' physical and mental well-being. As we know, the investigation of sleep problems for children and adolescents is relatively limited in China up to now. In the present study conducted in a large sample of primary and middle school students in Fujian Province, China, we explore whether sleep health (sleep duration, sleep disturbance, subjective sleep quality) was associated with behavioral, social, and physical factors to provide some reference value for suggestions for their sleep health in the future.

We observed that factors like sex, age, types of school, and regional differences suggest the need for tailored sleep promotion recommendations for children and adolescents with different characteristics. Boys had higher sleep health scores and lower sleep quality than girls, which is in the line with that shown in the previous studies (12, 13). The older the age in our study, the higher the sleep score, where a higher score means worse sleep. Some biological factors such as reduced homeostatic sleep pressure and delayed circadian rhythms in conjunction, as well as early high school start times and psychological changes from childhood to adolescence, might account for the differences in sleep patterns among children and adolescents (14, 15). Elementary and junior high school students living in urban areas had more adverse sleep than those living in rural areas. Urban students have more night activities compared to rural students because of social and economic developmental disparities, which might result in reduced sleep duration for urban students and have a negative impact on sleep (16). The types of school differences in children and adolescents are also indicated in this study. A 12-country study demonstrates that experiencing some or a lot of fast-paced and highly competitive school environment (in comparison to none or a little school pressure) was associated with a decrease in sleep duration of 4.56 min per day and an increase in sleep onset difficulties of 0.32, respectively (17). Based on this study as well as previous research, we speculate that the difference may be due to students in key schools suffering from a high academic burden and test stress, placing them under a high level of psychological stress that may aggravate the sensitization of the sleep system and lead to heightened alertness, physiological arousal, and difficulty in falling asleep (18).

It is known that lifestyles have an effect on sleep health. In the current study, unhealthy living and behavioral characteristics include excessive daytime sleepiness, unhealthy sleep habits, eating before sleep, social media usage (screen time per day during school, frequency of using electronics 30 min before bedtime, using electronic products in the bedroom), and co-sleeper negatively impacting on sleep health.

Our study result shows that activities related to electronic products or media exposure before bedtime increased the incidence of sleeping problems in children and teenagers and found that one in five adolescents or children were classed as very high users, the crowd of using electronics 30 min before bedtime everyday. With the popularity of electronic products or media, the proportion of users who own mobile phones increased, which has caused a series of critical health problems (19–21). Accumulating studies for the past 10 years suggested that students who had used electronic products or inculcated media habits before bedtime had made easily sleep health worse than others (12, 22, 23). Consistent with these studies, Akçay demonstrated that as teenagers in Konya High School spent more time on their social media, their sleep quality become progressively worse (24). The decrease in self-reported sleep duration was also noted among U.S. adolescents from 2009–2015, which correlated with new media screen time (25). More than 50% of adolescents in our study had electronic products or media such as computers, iPad, cellphone, or television in their bedrooms. Continente demonstrated that adolescents with media devices in their bedrooms were more likely to be short sleepers (26). Through electronic products or media (computer, Internet, smartphone, et al.) usage, teenagers often play games, watch movies, TV series, or entertainment shows, chat online, or do other activities. These activities, a stimulus for brain stimulating neurons contributed to increasing the occurrence of adverse sleep outcomes in students. Moreover, as long time use a screen at night, the eyes of students are exposed to strong light, and its blue spectrum has a strong and specific effect on the retina (27). Light can not only prevent the secretion of melatonin leading to circadian rhythm disorder but also cause the desire of awakening to delay sleep (28). Besides, some adolescents reported having difficulties disengaging from social media to sleep (29).

About 19.9% of the current sample had co-sleeper, which associate negatively with sleep healthSince co-sleepers may do more activities, such as talking and playing before going to bed or make sleeping environment noisy, sleep problems are prone to come out easily in students sharing bed or room (30), which makes it easier for students to fall into the unhealthy habit of sleep. Of course, this result was influenced by the age of the participant. Younger people are likely to have co-sleepers because of parental care. With the increase of age, the number of co-sleepers decreases, but some co-sleeping occurred due to the family's economic situation. Meanwhile, our results also show unhealthy sleep habits such as getting up late in the morning, staying up late, and doing energetic exercise before bed, were related to sleep health. Furthermore, excessive daytime sleepiness is related significantly to sleep health. We suspect if a student is drowsy during the day, there will be fragmentation of night sleep and increased sleep pressure, which will affect night sleep. Our findings suggest that food intake preceding sleep has a negative impact on sleep health, consistent with previous studies (31, 32). A previous study showed snacking and drinking caffeinated beverages before going to bed were associated with significantly short sleep duration and poorer sleep quality (OR = 1.49 and 1.83, respectively) (33).

Relationships with families and peers also appeared to play a pivotal role in sleep health. Because home and school are the two places in which students' lives are mainly involved, students who received more social support from family and friends reported greater life satisfaction. According to previous research, for children and adolescents, higher psychosocial well-being was linked to longer nocturnal sleep duration and lower levels of sleep disturbances (34). In our study, students who are satisfied with their family life/classmates relationships sleep better. The hypothalamic-pituitary-adrenal axis was activated with the release of hormones that affect sleep architecture due to enduring stress (35) which in childhood can emerge from various sources like problems with the family and peers (36).

In the current study, fluctuation of emotions is an important factor for unhealthy sleep, which is consistent with previous studies (37, 38). Emotion and sleep are closely linked and many studies have shown that heightened emotional reactivities including strong positive and negative emotions are related to the maintenance of insomnia symptoms (39). According to previous studies, affective processes mediate the effect of cognitive and autonomic hyperarousal on sleep (40). Sleep problems are confirmed to be associated with decreased levels of vagal suppression, which is considered indices of emotion regulation. Additionally, higher levels of emotional intensity tied in with reduced sleep duration and increased nocturnal activity (41). However, the relationship between mood and sleep is complex and bidirectional because poor emotions can worsen sleep and vice versa (42), further research is needed.

To data, comparable data on sleep deprivation among children and adolescents in Fujian is limited, but our study adds relevant research evidence in this aspect. Furthermore, another strength of the current study is a Cross-sectional study of a relatively large sample size and comprehensive assessments of a series of sleep health variables, which make us research the potential factors influencing sleep. However, several limitations should be noted. The first is that causality will be difficult to demonstrate in a cross-sectional design, although there are multiple theories to support our findings. Second, the current research does not include factors such as family structure and socioeconomic status, school timetable, and parents' sleeping habits, which may be significantly related to the sleep health of Chinese students. Moreover, as a result of graduation having heavy academic stress, the last-year students (grades 6, 9, and 12) were not included in investigation. And we haven't considered the effect of students taking sleeping pills for the time being and have not excluded it. Although the sampling method takes into account regional economic development, however, the randomly selected areas appear to be unevenly distributed geographically.

## Conclusions

Overall, this study found that a series of factors are significantly associated with adverse sleep outcomes among children and adolescents. The results of the study have valuable clinical significance as our findings indicated that the existing sleep problem in children and teenagers could be, at least partly, intervened by reducing the use of electronic products before bedtime, especially cellphone, by avoiding mood swings frequently and by improving the routine habit before bedtime. Based on our results, we recommend the combination of school with family intervention, and parents, teachers, health professionals, and students should raise awareness of developing healthy sleep habits in usual life, which may be more effective in improving students' sleep health.

## Data availability statement

The original contributions presented in the study are included in the article/Supplementary material, further inquiries can be directed to the corresponding authors.

## Ethics statement

Written informed consent was obtained from the individual(s), and minor(s)' legal guardian/next of kin, for the publication of any potentially identifiable images or data included in this article.

## Author contributions

Designed and modified the manuscript: SWe and SWu. Designed the research and participated in the experimental design, coordinated and drafted the manuscript, data collection, achievement interpretation, and manuscript writing: XX, FZ, YC, JL, and ZZ. Analyzed the data: XX and YC. All authors have read and agreed to the published version of the manuscript.

## Funding

This research was funded by Joint Funds for the Innovation of Science and Technology, Fujian Province (2018Y9089), the Natural Science Foundation of Fujian Province (2019J01315), Professor Development Fund Project of Fujian Medical University (JS15002), Investigation and comprehensive exploration of intervention strategies on obesity and nutritional status of primary and middle school students in Fuzhou (2019B011), and Fujian Provincial hospital-high level hospital construction program (2018-GSP-001).

## Conflict of interest

The authors declare that the research was conducted in the absence of any commercial or financial relationships that could be construed as a potential conflict of interest.

## Publisher's note

All claims expressed in this article are solely those of the authors and do not necessarily represent those of their affiliated organizations, or those of the publisher, the editors and the reviewers. Any product that may be evaluated in this article, or claim that may be made by its manufacturer, is not guaranteed or endorsed by the publisher.
