# Supplementary material for: Sleep health and its related influencing factors in primary and middle school students in Fuzhou: A large multi-center cross-sectional study
Source: Front Public Health. 2022 Aug 4;10:924741. doi: 10.3389/fpubh.2022.924741 (PMC9386349; doi:10.3389/fpubh.2022.924741)
Supplement: Supplementary file 1 [file Data_Sheet_1.docx]

**Supplementary Online Content**

Sleep health and its related influencing factors in primary and middle school students in Fuzhou: a large multi-center cross-sectional study

# Supplementary Tables

Table S1. Sleep self-made questionnaire project structure.

Table S2. Factor loading of each item in sleep self-made questionnaire.

Table S3. Convergent validity and discrimination validity of sleep self-made questionnaire.

**Table S1. Sleep self-made questionnaire project structure**

| Dimension | Item | Option | Score |
| --- | --- | --- | --- |
| Sleep  duration  (4-16) | What time do you go to bed on weekdays？(1-4) | 20:00-22:00 | 1 |
|  |  | 22:00-24:00 | 2 |
|  |  | 0:00-2:00 | 3 |
|  |  | After 2:00 | 4 |
|  | What time do you go to bed on weekends？(1-4) | 20:00-22:00 | 1 |
|  |  | 22:00-24:00 | 2 |
|  |  | 0:00-2:00 | 3 |
|  |  | After 2:00 | 4 |
|  | How many hours do you sleep on weekdays? (1-4) | Less than 5:00 | 4 |
|  |  | 5:00-6:00 | 3 |
|  |  | 6:00-7:00 | 2 |
|  |  | More than 7:00 | 1 |
|  | How many hours do you sleep on weekends? (1-4) | Less than 5:00 | 4 |
|  |  | 5:00-6:00 | 3 |
|  |  | 6:00-7:00 | 2 |
|  |  | More than 7:00 | 1 |
| Sleep disturbance (2-12) | Do you have nightmares while sleeping? (1-4) | Not during the past month | 1 |
|  |  | Less than once a week | 2 |
|  |  | Once or twice a week | 3 |
|  |  | Three or more times a week | 4 |
|  | Is there any apnea or awakening while sleeping? (1-4) | Not during the past month | 1 |
|  |  | Less than once a week | 2 |
|  |  | Once or twice a week | 3 |
|  |  | Three or more times a week | 4 |
|  | Will you wake up more than 2 times a night? (0-1) | No | 0 |
|  |  | Yes | 1 |
|  | Is it difficult to fall asleep during waking up at night? (0-1) | No | 0 |
|  |  | Yes | 1 |
|  | Do you often feel that you can't move when you wake up? (0-1) | No | 0 |
|  |  | Yes | 1 |
|  | Do you feel physical pain during sleep? (0-1) | No | 0 |
|  |  | Yes | 1 |
| Subjective sleep quality (1-7) | Did you have a good sleep? (0-1) | No | 0 |
|  |  | Yes | 1 |
|  | How long have you been sleepless? (1-5) | Less than once month | 1 |
|  |  | 1-6 months | 2 |
|  |  | 6-12 months | 3 |
|  |  | 1-5 years | 4 |
|  |  | ＞5 years | 5 |
|  | Do you feel lack of sleep? (0-1) | No | 0 |
|  |  | Yes | 1 |

**Table S2. Factor loading of each item in sleep self-made questionnaire**

| Sleep duration | | Sleep disturbance | | Subjective sleep quality | |
| --- | --- | --- | --- | --- | --- |
| Item | Factor loading | Item | Factor loading | Item | Factor loading |
| What time do you go to bed on weekdays？ | 0.736 | Is there any apnea or awakening while sleeping? | 0.592 | Did you have a good sleep? | 0.794 |
| How many hours do you sleep on weekdays? | 0.727 | Do you often feel that you can't move when you wake up? | 0.581 | How long have you been sleepless? | 0.745 |
| What time do you go to bed on weekends？ | 0.716 | Will you wake up more than 2 times a night? | 0.578 | Do you feel lack of sleep? | 0.495 |
| How many hours do you sleep on weekends? | 0.632 | Do you feel physical pain during sleep? | 0.490 |  |  |
|  |  | Do you have nightmares while sleeping? | 0.468 |  |  |
|  |  | Is it difficult to fall asleep during waking up at night? | 0.460 |  |  |

**Table S3** Convergent validity and discrimination validity of sleep self-made questionnaire

|  | Sleep duration | **Subjective sleep quality** | **Sleep disturbance** |
| --- | --- | --- | --- |
| What time do you go to bed on weekdays？ | **0.816** | 0.362 | 0.215 |
| What time do you go to bed on weekends？ | **0.637** | 0.252 | 0.184 |
| How many hours do you sleep on weekdays? | **0.842** | 0.346 | 0.243 |
| How many hours do you sleep on weekends? | **0.540** | 0.173 | 0.171 |
| Do you have nightmares while sleeping? | 0.132 | 0.210 | **0.641** |
| Is there any apnea or awakening while sleeping? | 0.141 | 0.167 | **0.541** |
| Will you wake up more than 2 times a night? | 0.085 | 0.159 | **0.467** |
| Is it difficult to fall asleep during waking up at night? | 0.115 | 0.209 | **0.248** |
| Do you often feel that you can't move when you wake up? | 0.141 | 0.189 | **0.492** |
| Do you feel physical pain during sleep? | 0.139 | 0.179 | **0.438** |
| Did you have a good sleep? | 0.245 | **0.718** | 0.239 |
| How long have you been sleepless? | 0.319 | **0.841** | 0.251 |
| Do you feel lack of sleep? | 0.340 | **0.626** | 0.252 |

Bold numbers are convergence validity, others are distinction validity.
